# 'The divorce program': gendered experiences of HIV positive mothers enrolled in PMTCT programs - the case of rural Malawi

**DOI:** 10.1186/1746-4358-5-14

**Published:** 2010-10-26

**Authors:** John Njunga, Astrid Blystad

**Affiliations:** 1 Centre for International Health, University of Bergen, Norway

## Abstract

**Background:**

For HIV infected mothers in developing countries, choosing to enroll in a prevention of mother to child transmission (PMTCT) of HIV program is supposed to represent the first step towards protecting their child from possible transmission of HIV from mother to child. Counseling and testing enable HIV infected mothers to learn about their status and to obtain the benefits of a PMTCT package. The study on which this article is based explored experiences of HIV positive women and their partners linked to Prevention of Mother to Child Transmission of HIV (PMTCT) programs in Chiradzulu district, Southern Malawi.

**Methods:**

A qualitative study using in-depth interviews (IDIs), focus group discussion (FGDs) and case studies was carried at two PMTCT sites. IDIs and FGDs were recorded and transcribed. The case studies involved a deeper inquiry into the past, present and situational factors of selected participants.

**Results:**

In a context of customary matrilineal kinship, matrilocal residence patterns and complete male absence from the PMTCT program, the demand by the PMTCT program for partner disclosure played up fears of rejection among men given accusations of infidelity by the wives' relatives. This situation led many men to abandon their families. Mothers enrolled in PMTCT programs hence faced not only the fear of transmitting the virus to their infants, but also the loss of income and support associated with a departed husband and the social disgrace of a ruined family. Community members referred to the PMTCT program as 'the divorce program'

**Conclusions:**

PMTCT programs may vary in effectiveness in different contexts unless they fundamentally respond to socio-cultural factors as lived out in communities they intend to serve. The PMTCT program in rural southern Malawi is a case in point.

## Background

One of the most tragic aspects of the HIV/AIDS infection is the possible transmission of the virus from an infected mother to her baby. Mother to Child Transmission (MTCT) occurs when HIV is passed on from an infected mother to her child during pregnancy, labour or through breastfeeding. In Sub-Saharan Africa alone 13.3 million HIV positive women of child bearing age live with HIV [1]. This figure represents 59 percent of the adult population living with HIV infection in the region. Such high HIV prevalence among women of child bearing age represents a huge latent risk for mother to child transmission of HIV [2]. Evidence suggests that most mother to child HIV transmission occurs late in the pregnancy, and that in the absence of any intervention, between 20 and 45 percent of the infants will become infected from their HIV positive mothers [2].

### MTCT and PMTCT

The documented cost effective methods for preventing MTCT have resulted in huge prevention campaigns (the prevention of mother to child transmission - PMTCT - of HIV programs) in countries with high HIV prevalence. Interventions that are commonly used to reduce the risk of transmitting HIV from an infected mother include the use of antiretroviral therapy to curtail vertical HIV transmission, and the use of modified infant feeding to prevent transmission through infected breast milk.

Major international guidelines on HIV and infant feeding were released in 2001 by an interagency task team on mother to child HIV transmission comprising WHO, UNICEF, UNFPA and UNAIDS [3]. These guidelines promote different infant feeding options for women with differing socio-economic status. In the guidelines, so-called 'formula feeding' is recommended for women for whom replacement feeding is considered 'acceptable, feasible, affordable, sustainable and safe'. In contrast; where formula feeding is not considered to be 'acceptable, feasible, affordable, sustainable and safe', HIV positive women are encouraged to exclusively breastfeed for the first six months of their child's life.

During the International HIV and Infant Feeding Consultation meeting held in 2006 in Geneva, the documented tragic outcomes of attempts at replacement feeding in low income contexts led to a shift in policy and a firm recommendation of exclusive breastfeeding as the way forward for HIV positive infant feeding mothers [4]. Ongoing debates on the global HIV infant feeding guidelines underscore the dilemma of how to balance the risks of HIV transmission resulting from breastfeeding and the risks of abstaining from breastfeeding when the ultimate aim is infant survival.

Partner disclosure is a central concept in most PMTCT programs. The challenges related to the mothers' voluntary or involuntary disclosure of their HIV positive status have been brought up as a central obstacle in studies on PMTCT. Few studies have explored in depth the local experiences with partner disclosure in a PMTCT context.

In the present study we explore HIV positive women and men's experiences with partner disclosure in the 'Prevention of Mother to Child Transmission of HIV' (PMTCT) programs in Chiradzulu district, Southern Malawi. The study results are contextualized in terms of local marriage and kinship organization. In the discussion we argue that without proper knowledge of local contexts and culturally sensitive health promoting approaches - in this case exemplified through WHO's PMTCT initiative - well-meaning health programs may represent a threat rather than support to the population that is targeted by the initiatives.

## Methods

Because of our contextual interests and the sensitive aspects of HIV/AIDS, a qualitative design was found appropriate in our attempt to explore mothers' experiences with PMTCT programs. Our research questions required a depth of response iterating mother's complex experiences related to infant feeding, as well as the significance and meaning given to such experience.

In-depth interviews (including key informant interviews), focus group discussions and case studies were chosen as approaches for the data collection. The researcher did not employ participant observation as a research method, but long term presence at the two chosen PMTCT clinics generated substantial knowledge based on daily observation and informal discussion with differently positioned actors. The study was carried out between June and October 2007.

### Recruitment of study informants

Purposeful sampling was employed in the study. This sampling strategy implies an intentional and systematic selection of informants with varying characteristics with reference to the topic of concern in search for patterns of response as well as for ambivalence and contradiction. This method of recruiting study participants implies that data generated from the study cannot be generalized to the wider population, but the material can have substantial relevance beyond the study informants. The main researcher (the first author of the paper) presented and discussed the content of the study, including rationale, objectives, methods and ethical principles with the PMTCT staff at the clinics. Together with the contact nurses at the two PMTCT clinics, potential women were identified from a PMTCT register - with close attention pertaining to informant diversity related to distance from the health facility, education, age, religion, marital status, parity and infant feeding method.

### In-depth interviews (IDI), focus group discussions (FGD) and case studies (CS)

Ten HIV positive mothers were invited to participate in in-depth interviews, of which one mother declined participation. Further, ten HIV positive men were approached for in-depth interviews, all of whom agreed to take part in the study. Groups of men and women of six per session took part in four focus group discussions, each lasting for about one hour. Towards the end of the data collection exercise, five HIV positive women - from among the nine recruited for in depth interviews - were recruited for case studies. The case studies consisted of longer interviews with substantial probing and thus facilitated substantial depth to the information that was revealed. Depending on the availability of the informant, subsequent visits were arranged for follow up inquiry. All the participants in the IDIs and the case studies were linked to the hospitals because of their HIV positive status and their participation in the clinic PMTCT program, while the participants in the FGDs were recruited from ordinary community men and women. All interviews were conducted in Chichewa, the national language in Malawi.

### Data analysis

Data were recorded with permission of informants and transcribed verbatim for analysis. Analysis of data was done manually using the principles of systematic text condensation as described by Malterud [5] This implies going through four steps: repeated review of the transcripts to gain a thorough sense of the overall content in the texts, identifying central meaningful units in the material, condensation of the content through a coding of the text, and finally creating categories that contain the condensed meaning of the main themes in the material. The theme most strongly emerging from the interviews was the disturbing implications of the PMTCT program's emphasis of partner disclosure of HIV positive results by the women.

### Ethics

The informants were informed about the voluntary character of the participation in the study, about the confidentiality principle and about their right to withdraw from the study at any point without any explanation. The study received permits from the Health research committee of the ministry of health, the Chiradzulu district health office and relevant community leaders.

### Limitations

Purposeful sampling was employed for recruiting study participants. This method of recruiting study participants implies that data were generated from a limited number of study informants, and the results can thus not be generalized to the wider population. Qualitative research material can, however, have relevance for contexts, in this case for PMTCT programs, far beyond the ones explored in a particular study. Subjectivity is inherent in both the data collection phase, in the analysis and in the write up phase. It is, however, the character of the personal encounter during the interview that facilitates the in-depth generation of data that is the strength of the qualitative approach.

## Results

### Social organisation of the study community

The community in which the study was carried out is matrilineal. This means that study results are context-specific and cannot be generalised to a wider population. The matrilineal system implies that kinship relations spring out of female generational ties, and involves a matrilocal residence pattern where a man moves to his wife's village where he builds a family home at the time of marriage. This matrilineal practice derives from the fundamental belief that women are the key reproducers of the family lineage. According to the practice, relatives trace their roots to a common ancestress or to a common 'breast' (*bele limodzi*), the source of their lineage [6]. When a suitor moves to a woman's village it ensures that his wife remains united with her brothers, sisters and parents who will help bring up children born in her family.

### Organisation of PMTCT services at study sites

When women come to the PMTCT clinic they go through a group-based pre-test counselling session. This session provides women with general information on HIV testing and on prevention of mother to child transmission. After the group-based counselling, the mothers receive a short individual counselling session where an HIV test is routinely offered by PMTCT nurse counsellors. Mothers who test HIV positive and have gone through post-test counselling are clinically staged using clinical symptoms with reference to the WHO staging system. Mothers in need of nutritional foods were in both programs provided with soya flour and 'plumpy nut', a fortified peanut butter paste with milk and vitamins.

As part of the PMTCT counselling, nurses encourage HIV infected mothers to disclose their status to partners and to persuade the partners to come for Voluntary Counselling and Testing (VCT). PMTCT nurses explain to the mothers that disclosure will open doors to support from partners, and will reduce the stress and the sense of loneliness that mothers often suffer as a result of non-disclosure. They also explained that partner testing promotes adherence to PMTCT infant feeding choice due to the husband's support, and thus enhances the preventive effort of the program.

### Partner disclosure and family disruption

All mothers in our study reported that they had disclosed their HIV status to their partners. All nine women who took part in in-depth interviews and case studies reported that their disclosure was met by denial and immense anger from their partners and with lack of willingness to relate to the information. Highly emotional outbursts like: 'Why did you test without telling me', 'I will not go for that test', 'I do not have that virus' paint a graphic picture of partner anxiety, fear and denial.

For some mothers disclosure was experienced as holding their spouse responsible for infecting them. One mother said; 'When I tested HIV positive, I wanted to disclose to my husband because I knew that it was him who infected me.' Another mother shared her emotional experience during the moment of disclosure to her husband: 'I waited until we ate supper and the children were asleep. When I started telling him . . . I could not control myself - I broke into tears'.

All of the nine mothers in the in-depth interviews/case studies reported that their families were disrupted after they had disclosed their HIV positive status to their partners, i.e. their partners abandoned them. When some of these women later remarried and again disclosed their status, four were again abandoned by their new partners. The case study below is illustrative of the stories told by the women:

### Case study: A vicious cycle of HIV, PMTCT, disclosure and family disruption

"Julita is a 30 year old mother of three children. She married in 1995. When she was pregnant with her second child, Julita was tested for HIV and was found positive. She disclosed her status to her husband as she had been strongly encouraged to do by the PMTCT program. Three weeks before her expected date of delivery Julita left home to stay with a relative who lived close to the hospital. When the baby was born, a message was sent to her husband; but the boy who was sent to deliver the message reported that Julita's husband was not at home. When Julita and her newborn baby returned home from the hospital, her husband was nowhere to be seen. Inside her home Julita found that her husband had moved out with his belongings. Soon rumors were rife that her husband was living in another village with a different woman. Efforts to reach him were unsuccessful. Julita accepted her fate and moved on with life taking care of her children.

A shop owner in the village (who was Julita's distant cousin) supported and provided for Julita and her children with money. Two years later Julita's child was tested and found to be HIV negative. At about the same time, the shop owner who had been supporting her family started seeing Julita more frequently. Their relationship developed and they soon moved in together. Everything seemed to be well until suddenly Julita fell ill; she had nausea and was vomiting. The same month she discovered that she was pregnant again. When she told her partner about the pregnancy, he appeared indifferent. Unfortunately, Julita became very weak, got diarrhea, persistent fever and lost weight. At this point, her partner left the village and relocated his shop to a village on the border of the district. At the time of the interview Julita had given birth again and was a member of the PMTCT program when we met her for this interview. She was unemployed and had no husband to support her."

Although every story told by the study informants varied, the story of Julita speaks well to the disturbing experience of all the women interviewed; everyone had, with encouragement from the PMTCT program, disclosed their HIV status, and informants in the individual interviews and case studies had upon disclosure experienced family break up. In the communities where this research was carried out, the PMTCT program was referred to as the 'family disruption program' (*pulojekiti yothetsa mabanja).*

Similar detailed information relating to each participant's experience with family break up was not collected during the focus group discussions, but became a central theme of discussion. Participants observed that one reason why many women do not enroll in the PMTCT program is that they are frightened that they will face family problems like they have witnessed among other program attendees. Instead of the support and adherence to exclusive infant feeding so strongly portrayed by the guidelines followed by the program staff, disclosure had led to ruined marriages. Increased knowledge of the reasons for the family disruption was gained through interviews with men.

### Men, disclosure and PMTCT programs

When confronted with the issue of abandoning their wives upon disclosing HIV positive status, male informants explained that they often felt powerless. In a matrilocal society men easily fall victim to stigma and hostility from the wife's family members when an HIV positive test is revealed. In a community where a man marries into his wife's family and onto their land, he is referred to as '*mkamwini*', which translates to 'guest' or 'stranger'. Women who disclose their HIV positive status to their mothers or brothers often provoke feelings of animosity against their husband. The husbands will be regarded as the *"'stranger' who infected their child and sister with HIV" *as one informant put it. Men explained that the trauma of dealing with an HIV positive result became too much to bear when the mother-in-law and brothers-in-law became involved. In a climate of animosity or outright hostility, men would find it easier to walk away and abandon their homes.

Communication dimensions of the disclosure of the HIV positive status by the women to their husbands were also related to the disturbing family outcomes by both the female and the male study participants. One man said:

"Women must change the way they communicate with their husbands when tested HIV positive . . . Most women will come home from the hospital furious and will shout at their husband for infecting them. Women should calm down and explain properly. I think they should be taught how they to communicate their HIV result to their husband . . . so the situation is handled without raising blood pressure for themselves and their husband."

It became clear during the in-depth interviews and case studies that men were virtually absent from the PMTCT program. The male informants indicated that PMTCT is a women's issue as it has to do with the mother and her child. One of them said:

"If you want to find PMTCT, you will find it at the antenatal section of the clinic...this is the place where women seek help on various issues of motherhood. What will people think of me if I a man went and sat down? This is not a section where you see men except if you are hospital staff."

The sudden encounter with the program through the event where their wives disclose their HIV positive status thus became a shocking confrontation with the PMTCT program according to the informants.

## Discussion

The persuasion or sometimes even demand for partner disclosure by the PMTCT programs in contexts where men are virtually absent from the PMTCT counseling and information sessions resulted in shock, fear and denial on the part of the men. This implied a challenging vantage point for dialogue and infant feeding collaboration. The subsequent hardships experienced by the husbands who found themselves accused of bringing the infection into the family increased the experience of hardship by the men. The consequence has been divorce and tragic family disruption, to the point where community members have started to refer to the PMTCT project as 'the divorce program'.

The finding that partner disclosure is associated with hardship adds evidence to previous studies that have shown that mothers fear for their families' social and economic future should they disclose their HIV status [7]. These findings, however, are not universal; studies carried out in other African settings maintain that partner disclosure brings about family support and improves adherence to infant feeding options [8].

Our research findings demonstrate the need for basic knowledge about the community in which a health promoting program is to be introduced, and a culturally sensitive or culturally competent health program with design approaches embedded in local realities. Cultural competency implies a capacity to function effectively and in line with culturally accepted behavior and beliefs, and that relates to the specific needs presented by the clients and their communities [9]. A so-called 'cultural competency model' is based on a "set of practices, policies, attitudes and behaviours that come together in a system, agency or program and enables that system, agency or program to work effectively in diverse cultural situations" [9]. A culturally competent program is thus one that is tailored to its 'target' community by being responsive to the spectrum of cultural factors that influence experience, behaviour and attitudes [10].

Experience has long since documented that health programs may be effective in one cultural setting, but may vary in effectiveness in a different context. When applied to a health program, cultural competency approaches ensure that services are provided in a fashion that reflects the needs of particularly situated people. Such approaches can also serve as tools for measuring program 'outcomes' in terms of their differential impact between cultures. The basic idea of the model is the explicit recognition that one-size-fits-all health programs cannot meet the needs of utterly diverse populations [11]. The cultural competency model is based on the assumption that differing 'cultural groups' have equal entitlement to benefits of health programs regardless of their 'otherness'. Cultural competence techniques thus go beyond mere cultural awareness or the respect for different cultural perspectives.

## Conclusions

Health programs, in our case the PMTCT program, may simply not deliver results unless they fundamentally respond to the wide spectrum of socio-cultural factors that are present and lived out in community life. More research is surely needed on the PMTCT program to see how different localities relate to the quickly shifting global infant feeding guidelines. The success of the PMTCT program, and other health promoting programs thus depend on their ability to integrate beliefs, values and practices of different cultural settings into the program design. If not, the program may easily prove to cause harm rather than good to the ones it intended to serve. The PMTCT program in Southern Malawi is a disturbing case in point.

## Competing interests

The authors declare that they have no competing interests.

## Authors' contributions

JN did the research data collection, analysis and interpretation He also drafted the manuscript (a complete thesis of which was submitted as partial fulfillment of the requirements for the award of a Master of Philosophy in International Health degree with the University of Bergen, Norway). AB made substantial contributions to the conception, design, analysis and interpretation of data. Both authors read and approved the final manuscript.
